# Medicine Residency Training Program during COVID-19: Qatari Experience

**DOI:** 10.11604/pamj.supp.2020.35.25005

**Published:** 2020-07-29

**Authors:** Mohamed Salem Nasrallah, Hassan Abbas Tawfik, Muna Taher Aseel

**Affiliations:** 1 Suez Canal University, Egypt,; 2 Weill Cornell Medical College,Qatar,; 3 Family Medicine in Primary Health Care Corporation, Qatar

**Keywords:** Family medicine, training, COVID-19, Qatari, program

## Abstract

Family medicine residency training programs should put in place COVID-19 action plan in conjunction with other clinical departments, employee health personnel, and infection control leads. Programs should develop coping strategies that balance resident and patient safety, resident wellbeing, clinical training services, and resident education.

## Commentary

The world currently become the greatest public health threat since the 1918 influenza pandemic because no one know exactly the scenario of its epidemiology and subsequent challenges imposed by its persistence. Family medicine training programs should be well prepared for expected future waves of the COVID-19 pandemic. Tailoring of circumstances and adjustment are needed to as the situation develops. That is why it is the suitable time to revise strategies balancing resident and patient safety, clinical training, and education [1]. On February 29, 2020, Qatar reported its first Qatari case of COVID-19 coming from Iran and by 15th of March 2020 the first local cluster happened. The state of Qatar, Through Hamad Medical Corporation, has developed the System Wide Incident Command Committee - SWICC to manage the crisis in the country. Residents provide the bulk of direct patient care in Hamad Hospitals; Family medicine residents would be among the frontline staff mobilized to manage the situation [2].

During the pandemic crisis and emergency plans, Residents´ safety, wellbeing and education were the top priority of the residency programs and institutions. So, more flexibility was ensured to accommodate resident training needs [3]. Medical education programs face a lot of challenges posed by the wide spread of COVID-19, including short staffing due to resident mobility and illness also quarantine measures, heavy workloads, and disruption of usual training and educational activities and planned schedules. Training programs all over the world are currently facing this initial surge of COVID-19 infections, so the need to plan for multiple expected waves of the disease [4]. Family medicine residency program in Qatar is an accredited ACGME-I program. Reflections on the COVID-19 experience, are essential component in international collaboration and spread of information, as we were approaching the last quarter of resident academic year, looking forward enhance knowledge and practical experience in resident training. The Family medicine residency program director and chief residents, working closely with the department of medicine and division of infectious diseases leads, began intense planning in early March 2020 based on prior MERS-CO experience, we recognized the importance of quick and sustainable response.

**Safety challenges for residents and patients:** during previous pandemics of viral illness, such as the global H1N1 influenza pandemic of 2009, physicians in training have reported high levels of exposure to circulating viruses, as well as low levels of adherence to work restrictions and personal protective equipment recommendations [5]. With increasing COVID-19 prevalence, health systems had to shift residents to help COVID-19 patients care demands. Family medicine residents were in first line defense in Qatar, they serve in variety of settings such as quarantine and isolation hospitals under umbrella of Medical education department with close collaboration with family medicine program. For resident safety a lot of things had been done such as training of resident regard COVID-19 case definition and swabbing techniques, training on infection control measures and pregnant residents exempted from COVID-19 services to complete their rotation through phone consultations. Another aspect which we apply is modification of normal program activities to comply with public health recommendations regarding social distancing, through stopping academic day didactic gatherings. For patient safety continuity of care clinics were stopped, these clinics are the primary clinical training in primary health care corporation - west bay health center serving patients according to ACGME-I requirements, shifting of those patients under faculty phone consultation clinics. For patient safety at quarantine and in isolation hospitals, residents were working in teams under close supervision of consultants from Hamad Genera Hospital and Knowledge update regard COVID-19 was ensured through emails, watts groups and SMS messages with answering fears and concerns regard the event.

**Resident education:** resident education is a priority, continuing educational conferences help maintain a feeling of normality, which programs have reported residents desire. COVID-19 also afforded rich case-based discussions for professionalism and medical ethics in an out-break setting such as public health exceptions to patient confidentiality, mandated isolation, and quarantine orders [6]. The COVID-19 pandemic itself has served as an educational opportunity for residents to learn about epidemiology, population health, systems-based care, and advocacy [7]. Our program´s regular teaching didactics in academic half day release sessions were suspended because of social distancing measures to prevent further spread of infection. It is replaced by online webinars through Microsoft Teams Application. More interaction had been made with other residents from other residency programs which affected positively the scholarly activity enhancing participation in case reports and publications.

**Mentorship and resident wellbeing:** institutional policies for resident wellbeing specially regard returning to work after illness vary depending on available resources. Programs should be familiar with their institution´s illness and COVID-19 testing policies. In general, residents with symptoms such as fever, cough, malaise, and myalgias should be excluded from work-related activities. Residents at risk of developing complications from COVID-19, such as those with immunosuppression or pregnancy, should be given an opportunity to confidentially contact program director with their concerns so that accommodations can be made to limit their exposure as much as possible. Finally, Trainees during early pandemic have reported high levels of stress and anxiety: programs should ensure mechanisms are in place for monitoring trainee emotional wellbeing [8]. Providing trainees with clear, consistent messaging is both challenging and important. Programs should consider developing a standardized format and frequency of updates to prevent confusion from information overload [9]. Family medicine training program distribute residents on faculty mentors, each faculty is responsible for 2-4 residents, the main role is to communicate with residents regard their needs during this vital period and report to program director.

One of the important early challenging stress regard transmission of infection from patients and ultimately to family members however by time and training residents were able to pass this distress, Actually no much concern were found regard working hours or leaves. The main issues raised were related to how they will get promoted and postponing of clinical and departmental examinations, and uncertainty about how other rotations will be affected in the long run. Family medicine training program in collaboration of Medical education department check in regularly with residents, especially those working at the frontline pandemic teams to rotate residents from pandemic team to non-pandemic team to decrease burnout and distress also Provide psychological support. Family medicine training program update residents regard changes in work environment and send them emails for reassurance regard promotion and compensation for missed rotations from elective periods. The program director, regularly communicate with residents and sending a message of solidarity and pride in our response efforts, also appreciation certificates had been distributed.

**Clinical training and promotion:** clinical services also become disrupted in COVID-19 spread due to the canceling of elective procedures and limiting of travel outside the home country. Educating trainees in the use of telehealth equipment, procedures, and etiquette is vital to ensure patients are still able to receive care [10]. Our residency program had to deal with residents based on clinical service rather than training needs, but not violating work hours, indefinitely postponing elective rotations, keep core rotations as possible as we can, and postpone examinations required for residency completion and promotion through clinical competency committee. For evaluation and assessment: Multiple Choice Questions(MCQ) and Objective Structured Clinical Examination which are considered for basis for annual summative evaluation and promotion were postponed but this made us to put an imagination of online exams and we had previous experience of MCQ preparation but resident attend at site, this time because of social distancing we tried to take complete online MCQ but this may be in September 2020. Extensive discussions had been made to practice online OSCEs and attending online webinars, but we found a lot of information technology aspects which made us to postpone, another suggestion was to make structured oral exam but because of situation also postponed. clinical competency meeting postponed to July 2020 and review of evaluations up to march including usual evaluation methods.

**Faculty members in COVID-19:** the challenge was to handle the expected surge of suspected and confirmed COVID-19 patients and maintain the department´s usual clinical services. Our planning also had to take into account the need for establishing COVID-19 teams in west bay health center - main training site, by these faculty members who were able to adapt most effectively to the situation offered positive role modeling to their residents. Faculty development program shifted from usual topics to COVID-19 case detection and swabbing, infection control measures procedures, teleconsultation principles and advantages, these webinars carried out by workforce training in primary health corporation. Virtual consultation clinics had been established first through telephone then video consultation replacing the usual clinical care provided in continuity of care clinic serving in its early phase the vulnerable risk groups by age and chronic illnesses.

Faculty members above age of 60 years with chronic illnesses were allowed to work from home, other faculty members were attending in teams every other day to apply social distancing, pregnant faculty members were allowed to work from home through call center related to Ministry of Public Health (MOPH) serving answering COVID-19 patients questions. Faculty members were volunteered to work with residents from early stage of pandemic in quarantine places supervising residents and serving essential patient care in these places, one the most volunteered work was participation with physicians from Qatar medical Association in psychological support for COVID-19 patients answering their concerns and support them. Faculty members were working in different things such as interviewing new resident patch for resident selection of family medicine training, others were completing their scholarly activity and publications while another group were participating in Qatari Board establishment committee.

## Conclusion

Resident wellbeing and psychological support should be the priority for all family medicine residency programs through establishing effective mentorship program. Clear communication and information update for residents and faculty members are considered corner stones in pandemic crisis management. Establishment of distant learning educational activities as back up method for resident training in case of second wave of the COVID-19 pandemic. Establish collaboration between programs on national and international academic levels for training purposes.

## References

[1] Anton M, Wright J, Braithwaite M, Sturgeon G, Locke B, Milne C (2020). Creating a COVID-19 Action Plan for GME Programs. Journal of Graduate Medical Education In-Press.

[2] Salameh M, Alhammoud A, Dosari MAAA, Alhaneedi GA (2020). The Orthopedic Residency Program at Hamad Medical Corporation during COVID-19 Crisis: An Evolving Educational Strategy. Research gate.

[3] Espana-Schmidt C, Ong EC, Frishman W, Bergasa N, Chaudhari S (2013). Medical residency training and hospital care during and after a natural disaster: Hurricane sandy and its effects. Am J Med.

[4] Leung K, Wu JT, Liu D, Leung GM (2020). First-wave COVID-19 transmissibility and severity in China outside Hubei after control measures, and second-wave scenario planning: a modelling impact assessment. Lancet.

[5] Perio MAD, Brueck SE, Mueller CA, Milne CK, Rubin MA, Gundlapalli AV (2012). Evaluation of 2009 pandemic influenza A (H1N1) exposures and illness among physicians in training. Am J Infect Control.

[6] Rakowsky S, Flashner BM, Doolin J, Reese Z, Shpilsky J, Yang S (2020). Five questions for residency leadership in the time of COVID-19. Acad Med.

[7] Liang ZC, Ooi SBS, Wang W (2020). Pandemics and their impact on medical training. Acad Med.

[8] Rambaldini G, Wilson K, Rath D, Lin Y, Gold WL, Kapral MK (2005). The impact of severe acute respiratory syndrome on medical house staff: a qualitative study. J Gen Intern Med.

[9] Poonia SK, Rajasekaran K (2020). Information overload: a method to share updates among frontline staff during the COVID-19 pandemic. Otolaryngol Head Neck Surg.

[10] Eichberg DG, Shah AH, Luther EM, Menendez I, Jimenez A, Perez-Dickens M (2020). Letter: academic neurosurgery department response to COVID-19 pandemic: The University of Miami/Jackson Memorial Hospital model. Neurosurgery.

